# Evaluating the Efficacy of Repurposed Antiretrovirals in Hepatitis B Virus Treatment: A Narrative Review of the Pros and Cons

**DOI:** 10.3390/ijms26030925

**Published:** 2025-01-23

**Authors:** Samuel Chima Ugbaja, Simon Achi Omerigwe, Saziso Malusi Zephirinus Ndlovu, Mlungisi Ngcobo, Nceba Gqaleni

**Affiliations:** 1 Discipline of Traditional Medicine, School of Nursing and Public Health, University of KwaZulu Natal, Durban 4000, South Africa; ndlovuS2@ukzn.ac.za (S.M.Z.N.); ngcoboM3@ukzn.ac.za (M.N.); 2 Strathclyde Institute of Pharmacy and Biomedical Sciences, University of Strathclyde, Glasgow G4 0RE, UK; sa.omerigwe@gmail.com

**Keywords:** antiretrovirals (ARVs), antiretroviral therapy (ART), drug repurposing, HIV, HBV

## Abstract

Human immunodeficiency virus (HIV) and hepatitis B virus (HBV) continue to be global public health issues. Globally, about 39.9 million persons live with HIV in 2023, according to the Joint United Nations Programme on HIV/AIDS (UNAIDS) 2024 Fact Sheet. Consequently, the World Health Organisation (WHO) reported that about 1.5 million new cases of HBV occur, with approximately 820 thousand mortalities yearly. Conversely, the lower percentage of HBV (30%) cases that receive a diagnosis is a setback in achieving the WHO 2030 target for zero HBV globally. This has necessitated a public health concern to repurpose antiretroviral (ARV) drugs for the treatment of HBV diseases. This review provides an introductory background, including the pros and cons of repurposing antiretrovirals (ARVs) for HBV treatment. We examine the similarities in replication mechanisms between HIV and HBV. We further investigate some clinical studies and trials of co-infected and mono-infected patients with HIV–HBV. The topical keywords including repurposing ARV drugs, repurposing antiretroviral therapy, Hepatitis B drugs, HBV therapy, title, and abstracts are searched in PubMed, Web of Science, and Google Scholar. The advanced search includes the search period 2014–2024, full text, clinical trials, randomized control trials, and review. The search results filtered from 361 to 51 relevant articles. The investigations revealed that HIV and HBV replicate via a common route known as ‘reverse transcription’. Clinical trial results indicate that an early initiation of ARVs, particularly with tenofovir disoproxil fumarate (TDF) as part of a regimen, significantly reduced the HBV viral load in co-infected patients. In mono-infected HBV, timely and correct precise medication is essential for HBV viral load reduction. Therefore, genetic profiling is pivotal for successful ARV drug repurposing in HBV treatment. Pharmacogenetics enables the prediction of the right dosages, specific individual responses, and reactions. This study uniquely explores the intersection of pharmacogenetics and drug repurposing for optimized HBV therapy. Additional in vivo, clinical trials, and in silico research are important for validation of the potency, optimum dosage, and safety of repurposed antiretrovirals in HBV therapy. Furthermore, a prioritization of research collaborations comprising of regulators and funders to foster clinically adopting and incorporating repurposed ARVs for HBV therapy is recommended.

## 1. Introduction

The Joint United Nations Programme on HIV/AIDS (UNAIDS) 2024 Fact Sheet estimated the 2023 global persons infected with human immunodeficiency virus (HIV) to be about 39.9 million [1]. HIV primarily targets the destruction of the CD4+ T cells (the immune system’s white blood cells). The destruction of CD4+ T cells weakens the ability of the body to fight various infections and diseases. Untreated HIV infection continuously destroys the body’s ability to fight diseases, thereby resulting in acquired immune deficiency syndrome (AIDS) at a later stage [2]. The drugs used for treating or managing HIV are classified as antiretroviral therapy (ART). Persons with HIV are often advised to combine therapy, known as a “treatment regimen”. The list of recommended Food and Drug Administration (FDA) HIV drugs has been previously reported in the literature and is shown in Table 1 below [3]. Another global viral infection called “viral hepatitis” accounts for over 80% of five different types of hepatitis such as hepatitis A, B, C, D, and E. Hepatitis B (HBV) and Hepatitis C (HCV) are regarded as the major causes of death from viral hepatitis. The World Health Organisation (WHO) reported that about 1.5 million new cases of HBV occur, with approximately 820 thousand mortalities yearly [4]. Conversely, the lower percentage of HBV (30%) cases that receive a diagnosis is a setback in achieving the WHO 2030 target for zero HBV globally [4,5]. Similar to HIV medications, drugs for the treatment of HBV-infected persons have been reported in the literature and are also shown in Table 1 below [6,7].

While ART involves the combinatory treatment (treatment regimen) of HIV with two or more antiretrovirals (ARVs), ARVs are medications or drugs used for the treatment of HIV. ARVs exist in different inhibitor classes, such as protease, non-nucleoside reverse transcriptase inhibitor (NRTI), nucleoside reverse transcriptase inhibitor (NNRTI), and other types [20,21,22]. Lately, ARV drugs have been repurposed or repositioned to treat HBV diseases [23]. Although drug repurposing and repositioning differ slightly, they have interchangeably been used to mean the same thing. Drug repurposing refers to using approved or existing drugs to treat diseases or conditions different from the originally approved purpose or condition.

Consequently, drug repositioning is using an approved drug for a different disease within the same or similar therapeutic class. In other words, drug repurposing involves using Food and Drug Administration (FDA)-approved drugs and repositioning for Non-FDA-approved conditions. For example, some drugs, such as tocilizumab, baricitinib, and remdesivir approved to treat other diseases, were repurposed to treat COVID-19 [24,25,26]. This study is aimed at investigating the potency of repurposed ARVs for treating HBV infections, exploring mechanistic similarities in the replication of HIV and HBV while assessing the pros and cons of selected repurposed ARVs for HBV treatment. It is essential to repurpose ARVs for HBV therapy because of the severe global impacts of HBV and the constraints of the current and approved HBV drugs including interferon therapies and nucleos(t)ide analogs. These common limitations include expensiveness, undesirable side effects, and common drug resistance. Conversely, the extensively defined and well-known safety profile of antiretrovirals is well documented. More so, the extensive accessibility, and two-pronged therapeutic approach for HIV and HBV is a less-costly option.

## 2. HIV and HBV Replication Cycle

**Figure 1:**
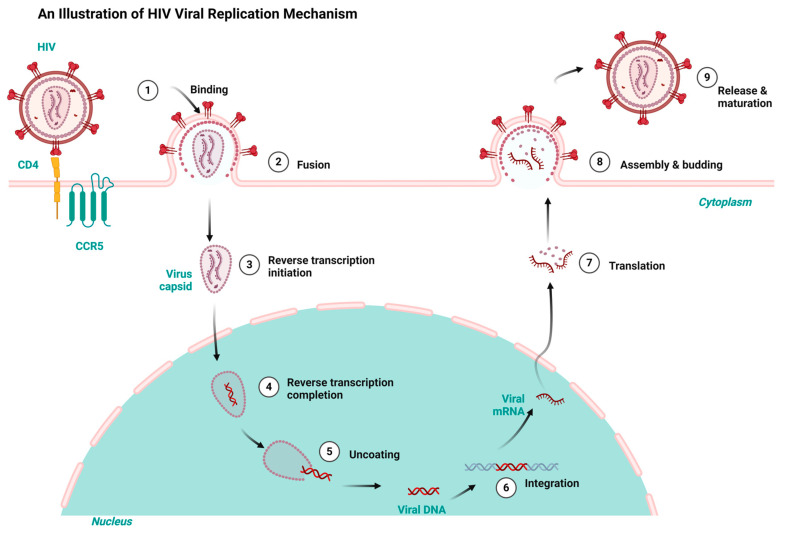
HIV replication cycle highlighting the key stages targeted by antiretrovirals for HIV treatment, redrawn with a bio-render as adapted from source [32].

**Figure 2:**
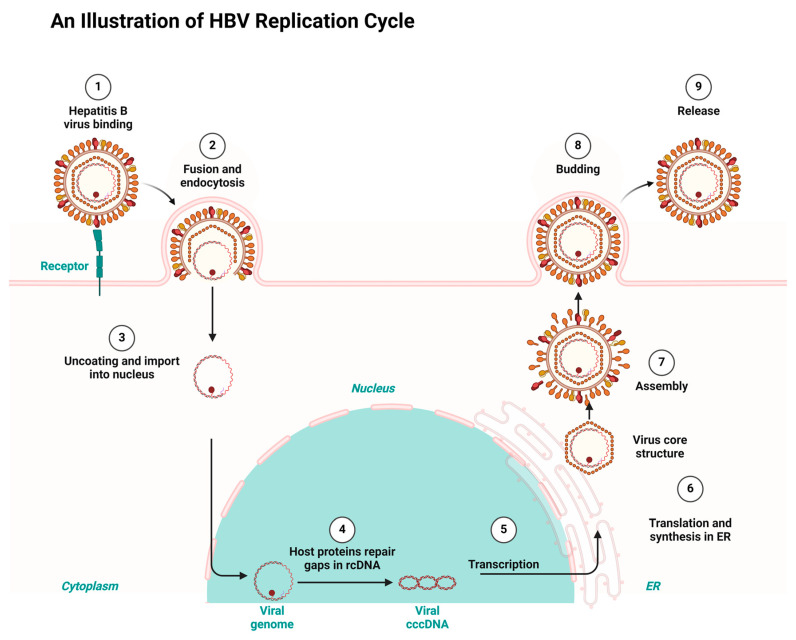
HBV replication cycle highlighting the key stages targeted by the antiretrovirals repurposed for HBV treatment, redrawn with a bio-render as adapted from source [33].

Having a good knowledge of the viral replication mechanisms used by both HIV and HBV is key to designing drugs for their therapy and repurposing and repositioning targets. HIV and HBV, as viral pathogens, replicate via a common route known as ‘reverse transcription’. However, HIV is a ribonucleic acid (RNA) virus and HBV is a deoxyribonucleic acid (DNA) virus. While HIV RNA transcribes to DNA using reverse transcription inside the host, HBV first transcribes to RNA and uses the same reverse transcription to return to DNA within the viral particles [7,27]. Consequently, the integration of HIV’s DNA into the genome of its host fosters it to replicate in the host’s system. Conversely, HBV integration takes place using genomic fragments, resulting in liver cancer [28,29]. Both HIV and HBV infection result in chronic infectious diseases but differ in their manifestation. Both HIV and HBV exhibit a persistent nature and maintain a latent reservoir in the host’s system. For example, HBV results in hepatocellular carcinoma and a cirrhosis of the liver, while HIV results in acquired immune deficiency syndrome (AIDS) [30,31]. Figure 1 and Figure 2 depict the HIV and HBV replication cycles.

HIV replicates when the virus attaches to the host organism’s CD4 and C-C Chemokine Receptor Type 5 receptors. The subsequent fusion of the virus with the cell membrane allows the entrance of the viral capsid into the cytoplasm, thereby initiating the reverse transcription and conversion of the viral RNA to DNA. The uncoating of the viral capsid releases the DNA virus into the host’s nucleus. The viral DNA is integrated into the host’s genome and transcribed into viral messenger ribonucleic acid (mRNA). The subsequent translation of the mRNA results in viral proteins that aggregate in the cell membrane and mature into infectious HIV virions that infect new cells, as shown in Figure 1 [32].

Consequently, HBV replicates when the virus fuses to a specific receptor on the host cell surface. The binding of the virus is followed by endocytosis, which allows the virus entry into the host. The endocytosis is followed by uncoating the viral capsid and attaching the viral genome to the host’s nucleus. HBV gains entrance to hepatocytes using a receptor-mediated mechanism which involves the sodium taurocholate co-transporting polypeptide (NTCP). HBV binds to the cell surface through heparan sulfate proteoglycans. A consequent attachment to NTCP enhances the viral entry, followed by the Fusion and uncoating. This allows HBV to transfer its relaxed circular DNA (rcDNA) into the host’s nucleus for replication. Existing gaps in the relaxed circular DNA (rcDNA) are repaired by the host’s proteins and converted into covalently closed circular DNA (cccDNA) in the template for transcriptions. The subsequent exportation of the transcribed RNA into the cytoplasm for the synthesis and translation into viral protein takes place at the endoplasmic reticulum (ER). Finally, the aggregation of the viral core structure around the new viral genome results in the maturation of new infectious viruses, which are released to infect new cells [33].

## 3. Clinical Studies and Trials of Co-Infected and Mono-Infected Patients with HIV–HBV

It has been established that ARV drugs primarily developed for HIV treatment have shown promise in managing HBV due to the overlapping pathways in viral replication. Further investigation into some key clinical studies and trials that demonstrate the efficacy of ARV drugs in HBV treatment, particularly in co-infected and mono-infected patients, is important [34].

### 3.1. Efficacy in Co-Infected Patients

Several studies have examined the role of ARV drugs in patients co-infected with HIV and HBV. The Strategic Timing of Antiretroviral Treatment (START) study initiated in 2009 was a multicentre international trial that involved over 1000 participants from 100 cities, 23 countries, and five continents [35]. It was revealed that ART can have effects on HBV replication with some ART regimens, including drugs that are effective against HBV, which can help manage the viral load of both HIV and HBV concurrently. While the primary outcomes of the START study are related to HIV progression, the secondary outcomes indirectly provided insights into how early ART may influence HBV-related conditions, particularly liver morbidity, as early ART initiation could have implications for liver health in HIV/HBV co-infected individuals. Results indicated that an early initiation of ART, particularly with tenofovir disoproxil fumarate (TDF) as a part of a regimen, significantly reduced the HBV viral load in co-infected patients. Therefore, findings from START could inform clinical guidelines for managing patients who are co-infected with HIV and HBV, particularly regarding the timing of ART initiation and the selection of appropriate regimens [35,36,37,38].

Furthermore, ACTG, A5175, and A5178 were studies conducted between 2003 and 2005, while A5394 was initiated in 2022 by The AIDS Clinical Trials Group (ACTG), which is a collaborative network involving various clinical research sites affiliated with the ACTG in the United States and internationally. The A5175 trial evaluated the efficacy of TDF in HIV/HBV co-infected patients; its data demonstrated that TDF was not only effective in suppressing HIV but also led to sustained reductions in HBV DNA levels, hence suggesting dual benefits for co-infected individuals [38,39,40]. The A5175 trial had a total of 1571 participants recruited from nine countries (Brazil, Haiti, India, Malawi, Peru, South Africa, Thailand, Zimbabwe, and the United States) comprising of four continents (Africa 34.7%, Latin America/Caribbean 29.5%, Asia 22.6%, and North America 13.3%) [41,42].

Conversely, another clinical trial, A5178, focused on timely ART initiation, comparing early treatment to deferred treatment in individuals with higher CD4 counts. This is crucial for maintaining immune function and preventing liver-related complications in co-infected individuals. This study recruited 1200 participants from more than seven countries (United States, Canada, Brazil, South Africa, Thailand, India, and various European countries) across five continents (North America, South America, Africa, Asia, and Europe). It was concluded that an early initiation of ART showed a significant reduction in the risk of developing AIDS and serious non-AIDS events compared to deferring treatment until CD4 counts fell below 350 cells/µL. Previous studies showed that participants with early ART had lower mortality rates, and a reduced HBV viral load compared to those who deferred treatment [43,44,45]. The ACTG A5394 has proposed to recruit participants with both chronic HBV and HIV for more studies on considerations for early phase clinical trials in the HBV cure, safety, tolerability, and impact of certain drugs in both chronic HBV and HIV [46,47,48].

Urvi Rana et al. (2021) analysed data from 2419 participants within the Canadian Observational Cohort (CANOC) collaboration. The study included individuals across British Columbia, Ontario, and Quebec. The author’s retrospective analysis of co-infected patients showed that 95% of these participants achieved virological suppression with ARV therapy. This result indicated high efficacy in managing both infections and showed no significant difference in virological suppression rates between HIV mono-infected and HIV–HBV co-infected patients [49]. While this study reveals some clinical trials such as START, ACTG, A5175, and A5178, it is essential to mention some of the limiting findings from the reviewed clinical studies including inadequate and relatively selective sample sizes, restricted special or geographical coverage, and participants selection biases which might have compromised the accuracy of the results. More so, there are uncertainties of employing these clinical trials to resource limited regions which could be due to poorly equipped public health facilities. A consideration of the above-mentioned limitations will ensure a relatively feasible repurposing of ARVs for HBV therapy.

### 3.2. Evidence in HBV Mono-Infected Patients

In a 5-year clinical trial conducted at five referral hospitals in South Korea, 192 patients were enrolled and observed for 240 weeks. Among the participants, 90 were entecavir (ETV) resistant and 102 Adefovir (ADV) resistant, with 91.2% of the total participants resistant to lamivudine. After the 240-week study, 78.6% of the participants achieved a virological response defined as serum HBV DNA levels < 15 IU/mL. Notably, the proportion of HBV DNA levels was higher in the ETV resistance group (84.4%) compared to the ADV resistance group (73.5%), although the difference was not statistically significant (*p* = 0.07) [50]. When only participants who adhered to the treatment were analysed, it was seen that 85.8% of them achieved HBV DNA < 15 IU/mL. This significant difference in favour of the ETV resistance group (92.7% vs. 79.8%, *p* = 0.02, and mean change in HBV DNA (from the baseline of 3.85 log10 IU/mL) indicated a reduction in the viral load among participants [50]. Similarly, Lim et al. (2019), in a randomized clinical trial from the above participants in two groups, compared TDF monotherapy (*n* = 50) and TDF with ETV combined therapy (*n* = 52) in patients with ADV-resistant HBV for 48 weeks, followed by TDF monotherapy for additional 48 weeks. Both treatment regimens were revealed to have a high viral load reduction potency. At week 96, some patients retained baseline resistance mutations, but none developed new mutations with the safety profiles of both treatment regimens being comparable [50,51].

In the Tenofovir Alafenamide (TAF) vs. TDF study in chronic Hepatitis B patients in the United States and other participating countries, findings suggested that TAF is effective in reducing HBV viral load, potentially offering advantages in terms of renal safety and tolerability as compared to TDF which is crucial for patients who may have underlying renal issues or are at risk for nephrotoxicity [52]. One hundred and seventy-four individuals with HBV exhibiting resistance to multiple antiviral agents (including lamivudine, entecavir, and/or Adefovir) and undergoing TDF monotherapy for at least 96 weeks were randomly assigned in a 1:1 ratio to either switch to TAF (*n* = 87) or continue TDF (*n* = 87) for 48 weeks. At the baseline, 84 in the TAF group and 80 in the TDF group had HBV DNA levels under <60 IU/mL, and after 48 weeks, the proportion of patients with HBV DNA < 60 IU/mL was 98.9% (86/87) in the TAF cohort demonstrating non-inferiority to the TDF cohort which had 97.7% (85/87). Additionally, the TAF group experienced significant increases in mean body weight and total low-density and high-density lipoprotein at week 48 relative to the baseline [52].

Feng et al. (2020) studied the RO7049389, an inhibitor targeting the assembly of HBV capsids, to evaluate its safety, tolerance, pharmacokinetics, and effects on the viral load in healthy participants. RO7049389 binds to the HBV core protein, inducing incorrect capsid assembly, suppressing viral replication, and depleting the functional core protein. The study demonstrated a rapid absorption and elimination of RO7049389. A greater than dose-proportional increases in plasma exposure were observed, particularly when administered with food, suggesting a potential for effective dosing strategies. Although this study primarily assessed safety and pharmacokinetics, preclinical data indicated that RO7049389 could significantly reduce HBV DNA levels [53].

## 4. Benefits and Barriers Associated with Repurposing ARV for HBV Treatment

Previous studies have established that the predisposition of HIV infections enhances the possibility of HBV infections [54,55]. Following the common mechanisms through which HIV and HBV are transmitted, the tendency of co-infection has been established. It has also been reported that the enhanced life span of persons with HIV due to ART results in HBV as a co-infection in most cases and results in the previously mentioned liver diseases [55]. Conversely, HBV endangers the potency of ART by exacerbating hepatotoxicity. HBV infection exacerbates hepatotoxicity by triggering cirrhosis, tissue hardness, sclerosis, and the damaging of hepatocellular tissue, which may further be worsened by an elongated usage of ARVs. Moreover, the poisonousness of mitochondria and fatty liver are caused by some antiretroviral drugs such as NRTIs, nucleosides, and nucleotides, exacerbating liver disease in HIV and HBV co-infected persons. Therefore, repurposing or repositioning therapy is imperative in this case of established co-infections for effective and efficient outcomes [56]. Clinical trials determining the ideal regimen for the treatment of HBV–HIV in HIV-infected persons are still ongoing. However, Adefovir dipivoxil, lamivudine, and interferon alpha are common FDA-approved drugs for treating HBV. Emtricitabine, pegylated interferon alpha, and tenofovir disoproxil fumarate (DF) are also used to treat HBV [55,57,58,59]. A Preference for any of these drugs depends on the infection level of the person with HIV–HBV, the individual’s choice, and the envisaged pros and cons of each of the drugs. Therefore, combined therapy is advised for HIV/HBV [55,60,61]. Among all the drugs, lamivudine has been strongly recommended for persons with a HIV–HBV co-infection due to its competitive inhibition potency against the nucleoside triphosphates naturally incorporated into the HBV DNA. A dose combination of lamivudine and an antiretroviral regimen has proven to be effective in inhibiting HIV replication in HIV–HBV co-infection cases.

Some studies have demonstrated that a long-term use of lamivudine exacerbates the chances of mutation in the active domain of the HBV polymerase gene [55,62,63,64]. Consequently, emtricitabine is another active drug for treating HIV–HBV co-infections; however, as a fluorinated derivative of lamivudine, it exhibits the same mutation and drug as lamivudine [10]. Afterward, a landmark achievement in the treatment of HIV–HBV co-infection was attained by the introduction of Adefovir. Adefovir was initially approved for the treatment of HBV and to remedy lamivudine resistance [65]. The subsequent approval of tenofovir disoproxil fumarate (TDF), a nucleic acid analog of Adefovir for the initial treatment of HIV, was also found to be active for HBV treatment [65,66,67]. However, the likely side effects of some of the ARVs and HBV drugs are mentioned in Table 1.

Following the challenges associated with general drug repurposing and ARV to HBV in particular, ensuring drug potency and efficacy with minimal adverse reactions is essential. To overcome these setbacks, healthcare providers are encouraged to adhere to management options such as periodic liver function tests, and optimal antiretroviral therapy selection by using hepatoprotections including antioxidants or combined-ARVs. Previous reports have shown that a prolonged treatment of HBV with ARVs results in mutation and drug resistance, which diminishes the drug’s potency and efficacy. The combination therapy of ARV drugs such as tenofovir and lamivudine exhibited potency in slowing HBV replication but exacerbated a resistance to lamivudine in persons with HBV experiencing a prolonged treatment [68,69]. The design of drug resistance to HBV compromises the achievement of repurposing ARV combinatory treatment because of the constant monitoring of the resistance profile involved. Another challenge of repurposing ARV drugs for HBV is dose-optimization. Pharmacokinetics and viral load are essential properties considered in persons living with HIV, which might differ in treatment for persons with HBV. Drugs such as tenofovir, designed for treating HIV, have also exhibited an effectiveness in suppressing HBV. Therefore, dosage modification is necessary to ensure optimal dosage in HBV treatment and to prevent subsequent liver diseases [70,71,72,73]. More so, there is the problem of cumulative toxicity in some persons treated with ARVs. Long-term usage of ARV (such as tenofovir) has been implicated in bone demineralization and nephrotoxicity, which are not amplified in persons with HIV as seen in HBV patients. Therefore, the long-term usage of repurposed ARV for HBV requires adequate monitoring to prevent the already mentioned side effects and ensure successful therapeutic effects [74,75]. The additional problems of adhering to treatment and handling side effects associated with repurposing ARV (such as fatigue, renal failure, and gastrointestinal upsets) to treat HBVs should be considered [76,77]. Some persons with HBV do not show symptoms of the disease. Therefore, a strict adherence to treatment is often neglected, even in cases of adverse side effects. This negligence results in a setback in achieving the suppression of the virus and subsequent HBV treatment. Continuous education, awareness creation, side effect monitoring, and support provision are essential for attaining ARV repurposing for the treatment of HBV [78,79,80,81]. More so, the process of clinical data collection and drug approval impedes the implementation of studies of the repurposed ARV drugs for HBV. Apart from the already approved drugs for HIV–HVB treatment, including tenofovir disoproxil and emtricitabine, there is a critical need to accelerate the data collection process, and the clinical trial investigations/approval on the safety and potency of new single drugs for HBV [82,83]. The list of currently approved/investigated ARVs repurposed for HBV treatment and shown in Table 2. Lastly, accessing ARVs for HBV treatment globally might encounter some setbacks in some low-income regions with a HBV endemic. There is a need to extend the global accessibility of ARVs to target HBV treatment in these low-income regions to ensure equitability in line with the United Nations’ development goals [84].

Dual therapy is highly recommended in low-income regions such as some Asian and sub-Saharan African countries where HIV and HBV are endemic and constitute public health challenges [111]. Dual therapy is beneficial in the public health sector because it allows for an easier management of HIV–HBV infections for healthcare providers, especially in low-income regions [111,112,113]. Targeting HIV–HBV infections, combinatory therapy economically reduces the need for a variety of drug regimens and reduces the resultant adverse drug effects. combinatory therapy enhances patients’ adherence to the treatment, thereby prohibiting the further development of HIV and reducing the associated liver diseases [114,115]. Clinically, a dual-therapeutic approach decreases the HBV reactivation risks in persons living with HIV who are on treatment. Finally, a dual-therapeutic approach aligns with UNAIDS and WHO targets of the improved and increased accessibility of ARTs in HIV and HBV endemic regions [116,117,118]. Drug resistance monitoring, therapeutic efficiency, and adherence are keys to a successful, long-lasting dual therapy. Therefore, dual therapy benefits co-infected persons and enhances a sustainable and scalable global public health system [119,120].

## 5. Identified Research Gaps Associated with ARV Repurposing and Recommendations

Repurposing the unapproved treatment of HBV with ARVs approved for HIV presents a potential research gap. Most of the previous and ongoing clinical trials focus on the potency of these repurposed unapproved HBV therapies rather than the safety of the HBV patients in the long run, including the associated bone and liver problems. Many of the available studies are focused on persons who are co-infected by both HIV and HBV, failing to address individuals suffering from only HBV. Additionally, the lack of the knowledge of precise and accurate dose-optimization and ARV-resistance profiles applicable to HBV treatment poses another gap for further investigation. Therefore, more clinical investigations are suggested for peculiar persons with a tendency for HBV single-treatment who exhibit adverse reactions to ARV therapies. Pharmacogenomics, pharmacogenetics, and demographical differences should be considered when designing safe, specific, and efficient repurposed therapies for viral infections [121,122,123].

The essential contributions of pharmacogenomics and pharmacogenetics in unveiling the therapeutic pathways for repurposing ARV for HBV treatment cannot be downplayed. Several genetically related concerns should be addressed to ensure effective and efficient drug repurposing. Variations in the drug-metabolizing process should be prioritized, considering that the metabolism of ARV takes place in the liver by specific enzymes such as cytochrome P450 (CYP450), resulting in the magnitude of the potency, efficiency, and toxicity in the bloodstream. The rate of metabolism is also affected by an individual’s genetic polymorphism, the existence of two or more distinct forms of a gene or alleles, resulting in dosage issues when used in HBV treatment. The adsorption rate of ARVs by the liver cells and transportation by drug transporters such as p-glycoprotein are essential in repurposing ARV for HBV treatment. Therefore, a dosage adjustment should be considered for an effective HBV treatment with ARV drugs. Another issue to consider when repurposing ARVs for HBV treatments is the adverse drug effects, which are also genetically associated. Pharmacogenetic profiling aids in predicting persons predisposed to adverse drug reactions, such as the histocompatibility complex, class I, B 57:01 (HLA-B*5701) gene, which is associated with hypersensitive effects to some ARV drugs. Genetic variations of drug-target receptors also determine the pharmacodynamic responses of people living with HBV treated with ARVs. More so, mutation on HBV poses resistance, affecting the potency of the repurposed ARVs. For example, when HBV polymerase genes rtM204V/I and rtL180M mutate, it results in a resistance to nucleoside and nucleotide drugs which reduces the potency of these repurposed antiretrovirals. The use of combinatory therapy is recommended to improve the therapeutic aim. Genetic profiling is essential in the potency and effectiveness of ARV efficacy. For example, HLA-B*5701 screening assists in preventing hypersensitivity in abacavir and CYP2B6 polymorphisms, affecting efavirenz metabolism and toxicity. Therefore, the above-mentioned markers facilitate in optimizing drug selection, reduces negative drug effects, and enhances therapeutic outcomes. Case studies in HBV treatment depict that the genetic screening for IL28B polymorphisms forecasts interferon treatment response and improves personalized therapeutic approaches and patient outcomes. Therefore, conducting genetic profiling to determine these differences would be helpful in precision medicine. Finally, pharmacogenomics and pharmacogenetics enable the prediction of the right dosages, specific individual responses, and reactions, resulting in successful ARV drug repurposing [124,125,126,127,128,129,130,131,132,133,134].

## 6. Conclusions

The repurposing of antiretroviral drugs for the treatment of HBV represents a promising avenue for addressing the dual challenge posed by HIV and HBV co-infection. As highlighted by the UNAIDS and WHO reports, the substantial global burden of both viruses underscores the urgent need for effective treatment strategies. In our study, we have elucidated the similarities in the replication mechanism of HIV and HBV, particularly the role of reverse transcription, which forms the basis for considering ARVs as viable therapeutic options for HBV. Clinical trials have demonstrated that an early initiation of ARV therapy, especially with TDF, can significantly reduce the HBV viral load among co-infected individuals. These findings emphasize the need for timely and appropriate therapeutic interventions, particularly with mono-infected HBV patients, where precise medication regimens are crucial for effective suppression. Summarily, the integration of ARVs into HBV therapy is a strategic response to a pressing public health issue. Continued research and clinical trials are vital to further elucidate the efficacy and safety of profiles of ARV medications in HBV treatment, ultimately contributing to the WHO’s 2030 goal of eliminating viral hepatitis as a public health threat. Furthermore, the role of pharmacogenetics in optimizing treatment outcomes cannot be overstated. We, therefore, recommend that before repurposing ARVs for HBV treatment, a genetic profiling of persons living with HBV is essential to enhance the efficacy of HBV treatment, minimize adverse effects, and improve the overall patient adherence. We therefore recommend further studied with a focus on the healthcare providers and patients’ compliance geared towards enhancing the healthcare facilities provided for the repurposed antiretrovirals for hepatitis B virus treatment. There should be a keen observation and an elongated monitoring of the pattern of viral resistance among HBV persons treated with the repurposed ARVs.

More so, real-world research is pivotal for the validation of preclinical and clinical trial outcomes for an easy assessment of the treatment potency within diverse populations. Furthermore, a consideration of feasible healthcare farcicalities and the economic effects of using dual therapy, especially among the low-income geographical areas, is essential. This equitable access is useful in informing the right decision among policy makers, ensure equity and fairness in line with WHO’s vision for the improved global management of HBV. Research efforts should be geared towards the assessment of real-time evidence, focusing on bridging the gap between controlled-trial environments and real-world clinical practices. Also, targeted studies on mono-infected HBV persons could improve therapeutic tactics for this subgroup. Finally, delving into specialized research areas such as the evaluation of long-term potency, optimization of therapeutic regimens, and the identification of pharmacogenetic markers for personalized treatment will further bridge the gap in improving patients’ adherence to repurposed drugs.

## Figures and Tables

**Table 1:** Commonly used drugs for HIV and HBV infections and their suspected side effects.

Serial Number	Drug Names	Target Infections	Likely Side Effects	References
1	Tenofovir Disoproxil Fumarate (TDF)	HIV and HBV	Renal failure, reduced bone mineral density, nausea, and diarrhoea	[8]
2	Lamivudine (3TC)	HIV and HBV	Nausea, headache, lactic acidosis	[9]
3	Emtricitabine (FTC)	HIV and HBV	Headache, gastrointestinal problems, hyperpigmentation and fatigue	[10]
4	Efavirenz (EFV)	HIV	Neuropsychiatric issues, rashes, and elevated liver enzyme	[11]
5	Dolutegravir (DTG)	HIV	Headache, insomnia, weight gain, hypersensitivity issues	[12]
6	Zidovudine (AZT)	HIV	Myopathy, nausea, vomiting, anaemia, neutropenia	[13]
7	Ritonavir/Lopinavir (LPV/r)	HIV	Gastrointestinal issues, elevated liver enzyme, lipodystrophy, elevated level of blood lipid (hyperlipidaemia)	[14]
8	Entecavir (ETV)	HBV	Fatigue, headache, dizziness, abdominal pain, and nausea	[15]
9	Tenofovir Alafenamide (TAF)	HBV	Headache, abdominal pain, renal and bone problems	[16]
10	Adefovir Dipivoxil (ADV)	HBV	Headache, weakness, abdominal pains	[17]
11	Pegylated Interferon Alfa-2a (Peg-IFNα2a)	HBV and HBC	Fever, flu, neuropathy, anaemia	[18]
12	Telbivudine (LdT)	HBV	Elevated creatine kinase, myopathy, peripheral neuropathy, fatigue and headache	[19]

**Table 2:** Currently approved or investigated ARVs for HBV treatment, including their mode of action and disadvantages.

Name of Drug	Mechanism of Action	Disadvantages	Class of Drug	Two-Dimensional Structures as Adapted from PubChem	References
Adefovir Dipivoxil (ADV)	Useful for lamivudine resistance patients. It functions by inhibiting HBV DNA polymerase.	Lower efficiency when compared to newer drugs and an increased resistance in prolonged usage which needs strict monitoring. It also has nephrotoxic effects.	Nucleoside Analog	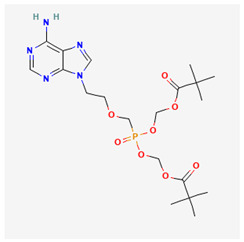	[85,86,87]
Tenofovir Disoproxil Fumarate (TDF)	Incorporates into the viral DNA resulting in the inhibition and premature termination of HBV DNA polymerase.	Prolonged usage results in renal and bone mineral problems. Caution and monitoring is also required for patients with associated kidney problems.	Nucleoside Analog	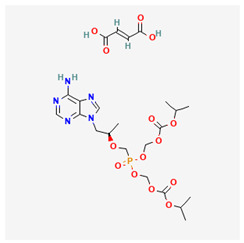	[88,89,90]
Lamivudine (3TC)	Terminates viral replication through the inhibition of HBV DNA polymerase.	It requires combined therapy and is only good for short-term use due to an increased resistance rate in prolonged usage in monotherapy.	Nucleoside Analog	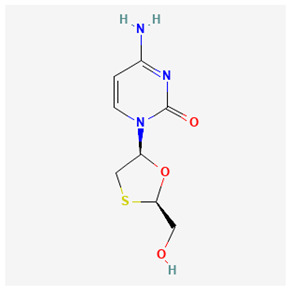	[91,92,93]
Emtricitabine (FTC)	Functions as a combined therapy with tenofovir for co-infection (HIV–HBV) by incorporating into viral DNA and preventing the replication.	Reduced potency due to cross resistance in a combined therapy with lamivudine.	Nucleoside Analog	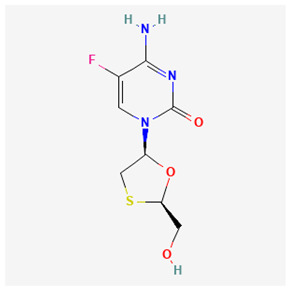	[10,94]
Entecavir (ETV)	Effectively terminates the HBV DNA polymerase chain resulting in an inhibition of the HBV replication.	Ineffective for HIV–HBV co-infection and not recommended for HBV patients with lamivudine resistance.	Nucleoside Analog	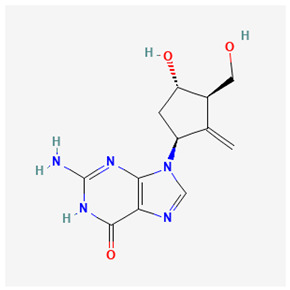	[95,96]
Nevirapine (NVP)	Inhibition of HBV reverse transcriptase and the disruption of viral DNA formation.	Increased risk of hepatotoxicity and accelerated drug resistance.	Non-Nucleoside Analog	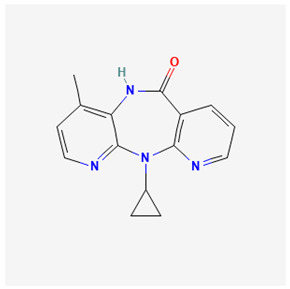	[97,98]
Efavirenz (EFV)	Incorporates into HBV polymerase and prevents the viral replication.	Neuropsychiatric side effects and reduced potency for HBV mono-infection.	Nucleotide Analog	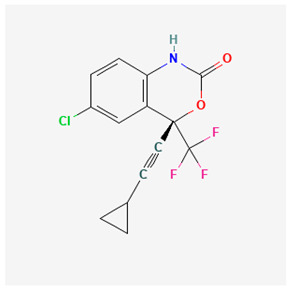	[99,100]
Tenofovir Alafenamide (TAF)	TAF is an improved brand of TDF. It inhibits HBV DNA polymerase replication.	TAF has kidney and bone problems, including other side effects in comparison to TDF.	Nucleotide Analog	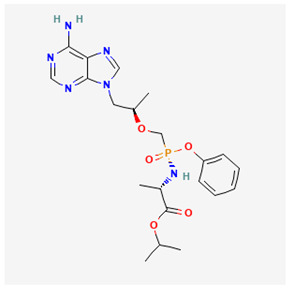	[101,102]
Atazanavir (ATV)	Inhibition of HBV protease-like activities and interference with viral maturation.	Functions effectively when boosted and has a tendency for increased liver enzymes.	Protease Inhibitor	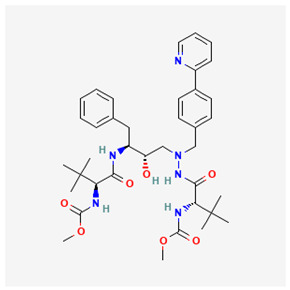	[103,104]
Lopinavir/Ritonavir (LPV/r)	Functions as a combinatory therapy for the inhibition of HBV replication, by modulating the host immune response.	Not originally approved as HBV drugs but has shown a level of potency in reducing the HBV DNA viral load. It has side effects, including gastrointestinal and lipid malfunctions.	Protease Inhibitor	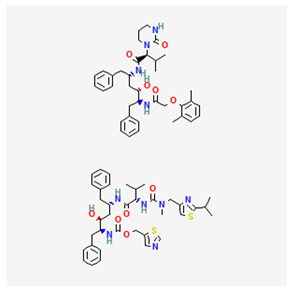	[105,106]
Raltegravir (RAL)	Inhibits the HBV DNA incorporation into the host genome thereby preventing replication.	Lower barrier to resistance and reduced clinical data in HBV.	Integrase. Strand Transfer Inhibitors	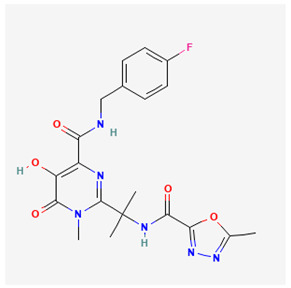	[107,108]
Dolutegravir (DTG)	Blocks HBV DNA incorporation and suppresses viral persistence.	Danger of hepatotoxicity and potential weight gain.	Integrase. Strand Transfer Inhibitors	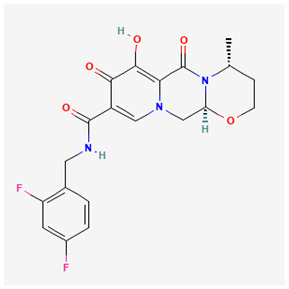	[109,110]

## Data Availability

Not applicable.
